# Supplementary material for: A Global Screen for Assembly State Changes of the Mitotic Proteome by SEC-SWATH-MS
Source: Cell Syst. 2020 Feb 26;10(2):133–155.e6. doi: 10.1016/j.cels.2020.01.001 (PMC7042714; doi:10.1016/j.cels.2020.01.001)

Q5ST30 | SYVM_HUMAN | VARS2 KIAA1885 VARS2L VARSL
Monomer MW [kDa]: 118.49 Monomer expected elution fraction: 39

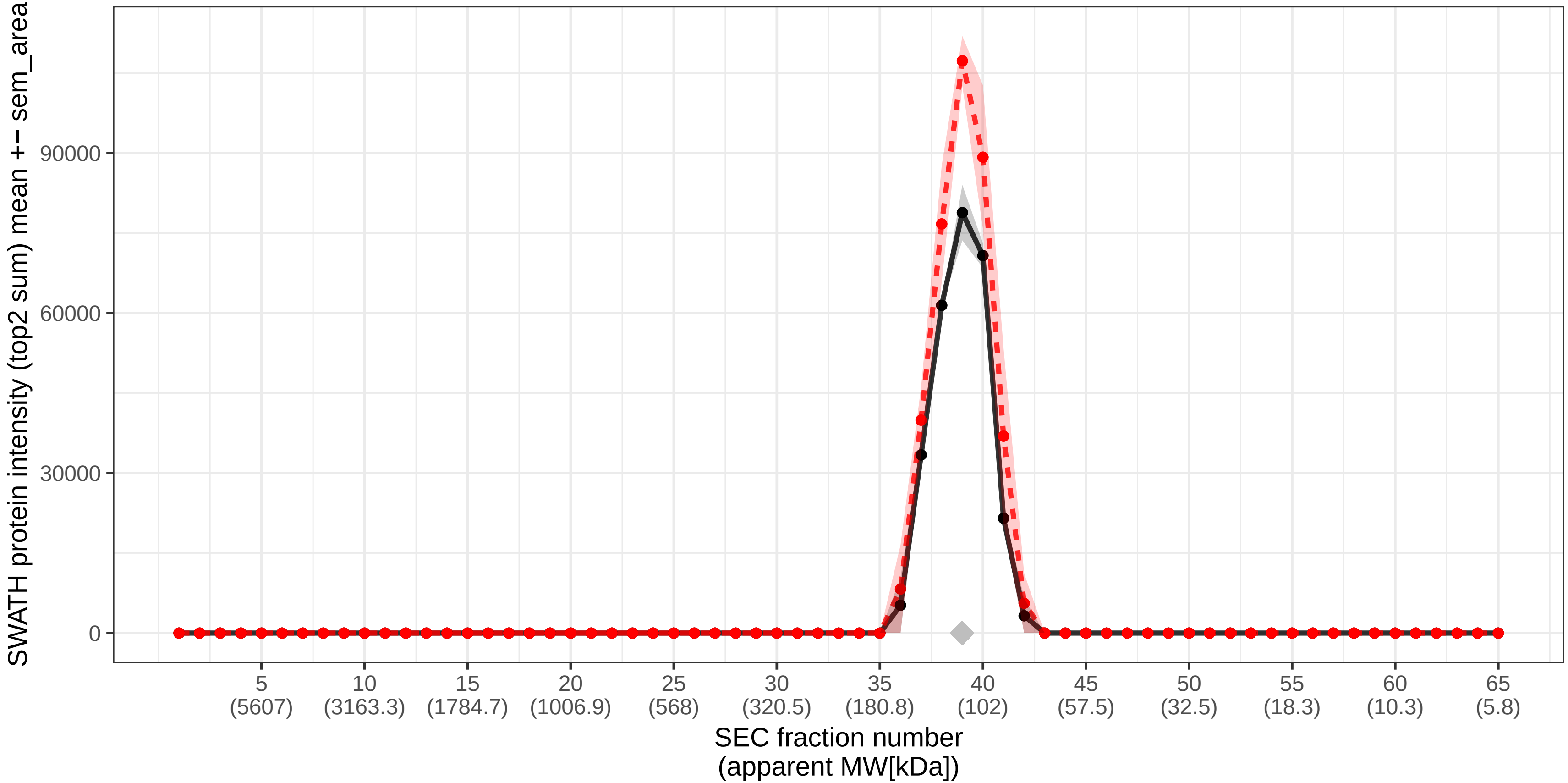

Supplement: Data S1. SEC-SWATH-MS Protein Chromatograms, Related to Figure 1 [file mmc6.zip › SECchrom_Q5ST30_SYVM_HUMAN_VARS2_KIAA1885_VARS2L_VARSL.pdf]
